# Case of Laceration of the Cervix Uteri, Consequent on Passive Labour

**Published:** 1822-05

**Authors:** Thomas Aspray

**Affiliations:** Licentiate of the Honourable Company of Apothecaries.


### OBSTETRICAL MEDICINE AND SURGERY.

### ART. VI.—*Case of Laceration of the Cervix Uteri, consequent on Passive Labour.* By THOMAS ASPRAY, Licentiate of the Honourable Company of Apothecaries.

I BEG permission to introduce to your notice the history of a case in midwifery, followed by a fatal issue; and, if it be worthy of your observation, and consistent with the design of your pages, I shall esteem its insertion in the ensuing Number of the London Medical and Physical Journal a particular favour.

Mrs. S—, aged thirty-seven years, of sanguine temperament and short stature, after the full complement of the term of gestation, was suddenly taken in labour of her sixth child, on March 22d, P.M. Being called to this case, and having examined, a combination of unfortunate incidents was found to form the presentation, which consisted of one hand, the vertex, and funis. The hand and funis were to the right side of the patient, and the vertex properly descending through the brim. At this period, such a concurrence of untoward circumstances (in a pelvis which had been found, in previous labours, encroached upon by an unusually large prominence of the sacrum,) was beyond rectification, because that the presentation had descended, under forcible uterine efforts, much beneath the brim of the pelvic cavity. From this time to the lapse of four or five hours, little progress was made: indeed, the pains suffered long intermissions, or were frequently of the metastatic kind. On this account, and from the stationary condition of the labour, a gentle friction and pressure of the abdominal parietes was practised for half an hour, with some farther passage of the head; but attempts to carry the funis and arm up beyond the brim were unsuccessful, from confinement of room, occasional severe pains, and consequent contraction. This gentle pressure had aided the uterine efforts so much, that the head was now within reach of the short forceps; and, as it remained here impacted for several hours, under a full and violent establishment of parturient effort, this instrument was proposed to the patient, as being likely to deliver her speedily from her distress and the occurrence of any evil, (as a laceration of the womb was feared,) but neither the advice of two professional men, nor that of those about her, although explicit as to the end proposed, and as to the danger of refusing a compliance, was availing, and the patient remained fatally opposed, through fear, as she observed, of suffering much pain; this impression we endeavoured, in vain, to convince her was false, as the application of the instrument could produce, when tenderly used, no unpleasant feeling. One hour and a half expired under the influence of this irresolute suspence; before the termination of which period, a forcible extrusion and repulsion of the placenta, with the emission of a few ounces of dark-coloured blood, occurred; and, immediately after, a sinking of the pulse, a cessation of pain, vomiting, and indeed a cadaverous hue prevailing over the features of the face, were perceived. At this time the patient consented to the use of the forceps; but, on examining, the head, &c. had retracted, so that the instrument could not now reach it: it was therefore deemed advisable, after a consultation, to extract the child manually, which had been destroyed, as regards its life, by pressure on the cord. From the complete recession of the head above the pelvis, after cutting away the placenta, the operation of turning was accomplished in about a quarter of an hour, although not without considerable difficulty, from hypertrophy of the head and general bulk. After this operation, although the uterus contracted kindly, the patient appeared sinking; when laudanum and brandy were administered in moderate doses, with warmth to the epigastric region and extremities, and friction used to the arms and legs, together with ventilated room and light bed-clothes: but means of this kind, persisted in, gained naught upon the mortal tendency, and she died in ten minutes or a quarter of an hour.

After her death, on again introducing the hand, a rupture of the cervix uteri, transversely at its posterior part, was found, from which accident blood had filled the vagina, and, partly, the abdominal cavity; yet the uterus had contracted so closely, that no more than the finger could be passed within it. This melancholy event had taken place some short time after the use of the forceps had been urgently proposed, and when it was probable that their use might have saved the life of the mother, and, perhaps, that of the child.

In this case, notwithstanding the head had descended to a sufficient proximity to the outlet to be commanded by the short forceps, it is clear it could not have arisen without violent uterine exertion, as the bones of the head had been so squeezed and pressed over each other as to resemble the figure of a cone with its apex at the vertex.

It is rather singular that, in this case of rupture, no sudden shriek occurred, or sensation of laceration, although numerous symptoms existed to conduct to a reasonable suspicion of the alarming event.

Does an accident of this character arise from any condition of the uterine structure at any peculiar period of labour, or in any notable constitution? or is the ordinary explanation, "of powerful action connected with unusual resistance," a satisfactory solution of the reason of the occurrence of rupture of the womb?

*Olney; March 30th,* 1822.

